# Gender- and Age-Dependent γ-Secretase Activity in Mouse Brain and Its Implication in Sporadic Alzheimer Disease

**DOI:** 10.1371/journal.pone.0005088

**Published:** 2009-04-07

**Authors:** Lisa Placanica, Lei Zhu, Yue-Ming Li

**Affiliations:** 1 Molecular Pharmacology and Chemistry Program, Memorial Sloan Kettering Cancer Center, New York, New York, United States of America; 2 Department of Pharmacology, Weill Graduate School of Medical Sciences of Cornell University, New York, New York, United States of America; New York State Institute for Basic Research, United States of America

## Abstract

Alzheimer disease (AD) is an age-related disorder. Aging and female gender are two important risk factors associated with sporadic AD. However, the mechanism by which aging and gender contribute to the pathogenesis of sporadic AD is unclear. It is well known that genetic mutations in γ-secretase result in rare forms of early onset AD due to the aberrant production of Aβ42 peptides, which are the major constituents of senile plaques. However, the effect of age and gender on γ-secretase has not been fully investigated. Here, using normal wild-type mice, we show mouse brain γ-secretase exhibits gender- and age-dependent activity. Both male and female mice exhibit increased Aβ42∶Aβ40 ratios in aged brain, which mimics the effect of familial mutations of Presenilin-1, Presenlin-2, and the amyloid precursor protein on Aβ production. Additionally, female mice exhibit much higher γ-secretase activity in aged brain compared to male mice. Furthermore, both male and female mice exhibit a steady decline in Notch1 γ-secretase activity with aging. Using a small molecule affinity probe we demonstrate that male mice have less active γ-secretase complexes than female mice, which may account for the gender-associated differences in activity in aged brain. These findings demonstrate that aging can affect γ-secretase activity and specificity, suggesting a role for γ-secretase in sporadic AD. Furthermore, the increased APP γ-secretase activity seen in aged females may contribute to the increased incidence of sporadic AD in women and the aggressive Aβ plaque pathology seen in female mouse models of AD. In addition, deceased Notch γ-secretase activity may also contribute to neurodegeneration. Therefore, this study implicates altered γ-secretase activity and specificity as a possible mechanism of sporadic AD during aging.

## Introduction

Alzheimer disease (AD) is a progressive and fatal neurodegenerative disease that is postulated to result from aggregation of toxic β-amyloid peptides (Aβ) [1], [2]. The vast majority of AD is sporadic in nature and has no known genetic basis [3]. Age is the most common risk factor associated with sporadic AD and the incidence rates of AD across all ethnicities increase exponentially with age, reaching 25 per 1,000 persons by age 85 [4]. Interestingly, gender is also an established risk factor as women have been shown to have a higher prevalence and risk for AD [5]–[10]. This apparent gender bias has also been recapitulated in multiple mouse models of AD as female mice exhibit more aggressive senile plaque pathology compared to male mice [11]–[15]. However, the molecular basis underlying this gender bias has not been fully resolved.

Overproduction and impaired clearance of Aβ can both contribute to increased plaque formation. Aβ peptides are produced by the sequential processing of the amyloid precursor protein (APP) by two proteases, β-secretase (BACE1) and γ-secretase [2]. Initial BACE1 processing of APP is required for subsequent γ-secretase mediated production of the Aβ40 and Aβ42 peptides that are the major components of amyloid plaques. APP can also be processed by α-secretase within in the Aβ domain and this cleavage precludes the formation of pathogenic Aβ peptides [16]. Numerous enzymes have been implicated in the degradation of Aβ peptides in the brain, including neprilysin, insulin-degrading enzyme, as well as endothelin-converting enzyme 1 and 2 [17]. The role of Aβ generating and Aβ degrading enzymes in aging and sporadic AD has begun to be investigated. Decreased neprilysin and insulin-degrading enzyme activity and expression have been associated with aging and increased activity of specific endothelin-converting enzyme 1 isoforms has been suggested to be protective against AD [18]–[20]. Elevated BACE1 activity has been found in both aged and AD brains and is associated with increased Aβ deposition [21], [22]. Reduced α-secretase activity in AD brain and reduced expression in the cerebral spinal fluid of AD patients has been reported [23], [24]. It is well established that mutations to presenilin (PS), the catalytic subunit of γ-secretase, are a causative factor in rare forms of hereditary early onset AD and result in an increased Aβ42∶Aβ40 ratio [3]. However, the role of γ-secretase activity in normal aging brain and sporadic AD has not been thoroughly investigated.

To determine the effect of gender and aging on *in vitro* γ-secretase, we examined its activity in C57BL/6 mouse brain from 1–24 month old mice. We found that both aging and gender significantly affect γ-secretase activity and specificity; male mice exhibited a sharp decline in brain APP γ-secretase activity with aging whereas female mice did not. Both male and female mice exhibited an increased Aβ42∶Aβ40 ratio in aged brain, indicating altered γ-secretase specificity during aging. The increased Aβ42∶Aβ40 ratio we detected in aged mouse brain is reminiscent of the increased Aβ42∶Aβ40 ratios seen in familial early onset AD and can also be correlated with the Aβ mediated plaque pathology in AD patients. Additionally, age was found to have a significant effect on Notch1 γ-secretase activity with both male and female mice having greatly reduced activity in aged brain suggesting altered substrate processing abilities with age. Therefore, our findings suggest a role for altered γ-secretase activity and specificity in aging and add to our understanding of the potential aberrant Aβ generating processes which play a role in the pathogenesis of sporadic AD and neurodegeneration.

## Results

### γ-Secretase activity exhibits age- and sex-dependent activity for APP and Notch 1 processing

**Figure 1:**
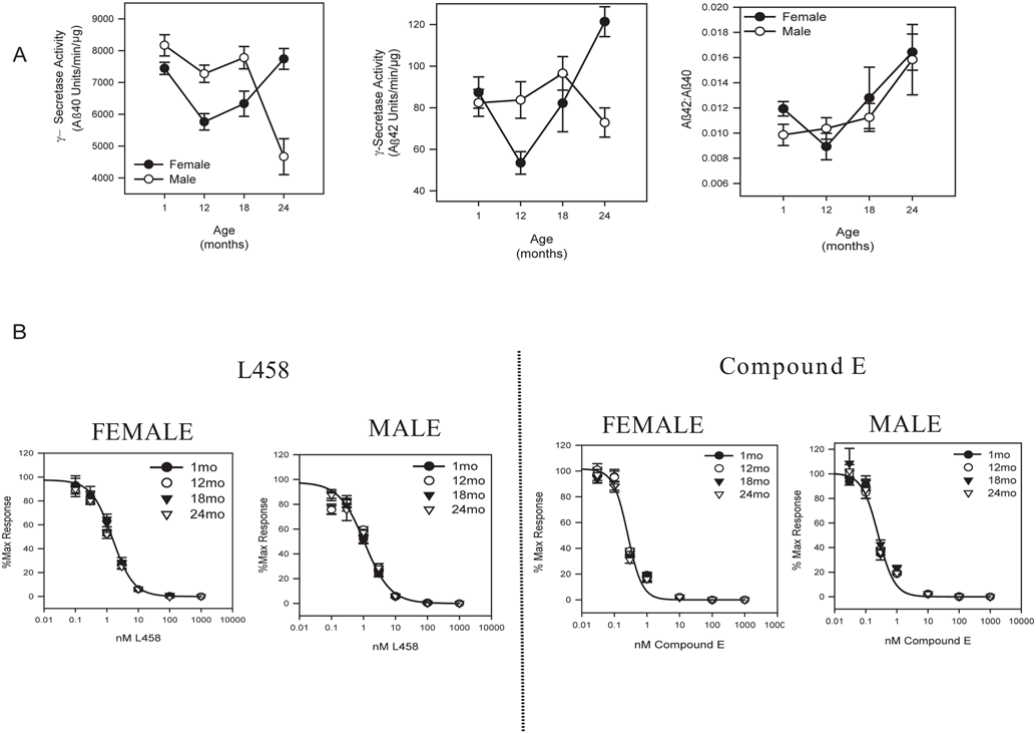
Age- and gender-specific APP γ-secretase activity in C57BL/6 mouse brain. (A) Male mice exhibit reduced γ-secretase production of Aβ40 and Aβ42 peptides in aged brain compared to females but both males and females exhibit increased Aβ42∶Aβ40 ratios. *In vitro* γ-secretase activity for Aβ40 (left) and Aβ42 (middle) production from total mouse brain was measured using a recombinant APP substrate. Background was defined as activity remaining in the presence of 1 µM Compound E. Values represent background subtracted ECL units/min/µg membrane (average±s.e.m., n = 16). The Aβ42∶Aβ40 ratio was calculated (right) (average±s.e.m., n = 8). (B) There is no effect of age or gender on sensitivity to distinct γ-secretase inhibitors. *In vitro* Aβ40 γ-secretase activity from total female and male mouse brain was measured using a recombinant APP substrate in the presence of increasing concentrations of L458 (left) or Compound E (right). The compound structures are indicated. Background was defined as activity remaining in the presence of 1 uM compound. Activity is plotted as a percentage of background subtracted maximal activity (% Max). Curves were generated using a sigmodial three parameter logistic fit in SigmaPlot8.0 (average±s.e.m, n = 4).

To determine if there was any age or gender influence on γ-secretase activity, we directly compared γ-secretase activity between male and female brains at varying ages. Total whole brain membrane fractions were isolated from male and female C57BL/6 mice at 1, 12, 18, and 24 months of age and *in vitro* γ-secretase activity was measured using a recombinant APP substrate. Aβ40 and Aβ42 were detected using G2-10 and G2-11 antibodies, respectively. Interestingly, male and female mice exhibited distinct activity profiles for both Aβ40 and Aβ42 production during aging (Figure 1A, left and middle panel, respectively). Male mice exhibited consistent Aβ40 production at 1, 12, and 18 months with 7500–8200 units/min/µg. However, at 24 months, male mice had approximately a two fold reduction in Aβ40 γ-secretase activity compared to 1 month, producing only 4500 units/min/µg (Figure 1A, left panel, open circles, p<0.00002). On the contrary, Aβ42 production in male mice was maintained at 70–95 units/min/µg during aging (Figure 1A, middle panel, open circles).

Although female mice had similar Aβ40 activity as males at 1 month, there was a transient 15–25% decline in Aβ40 activity in female mice at 12 and 18 months but by 24 months Aβ40 activity had returned to the 1 month baseline levels (Figure 1A, left panel, filled circles). Female mice therefore exhibited 65% more Aβ40 production compared to male mice at 24 months of age (p<0.00008). Similar to Aβ40 activity, there was a transient decline in Aβ42 production in female mice at 12 months but Aβ42 activity levels increased at 18 and 24 months resulting in a 30% increase in Aβ42 activity at 24 months compared to 1 month (Figure 1A, middle panel, filled circles, p<0.004). Compared to male mice, female mice had 68% more Aβ42 activity at 24 months of age (p<0.001). Both female and male mice had a similar increase in the Aβ42∶Aβ40 ratio with aging, with a 40% and 60% increase at 24 months compared to 1 month, respectively (Figure 1A, right panel, p<0.01 female, p<0.057 male).

We next examined γ-secretase sensitivity to two structurally distinct inhibitors, L685,458 (L458) and Compound E. Aβ40 *in vitro* γ-secretase activity was measured in the presence of increasing concentrations of either L458 (Figure 1B, left panels) or Compound E (Figure 1B, right panels). There was no difference in efficacy or potency of either L458 or Compound E in either females or males. Furthermore, there was no age related differences in efficacy or potency for either compound.

**Figure 2:**
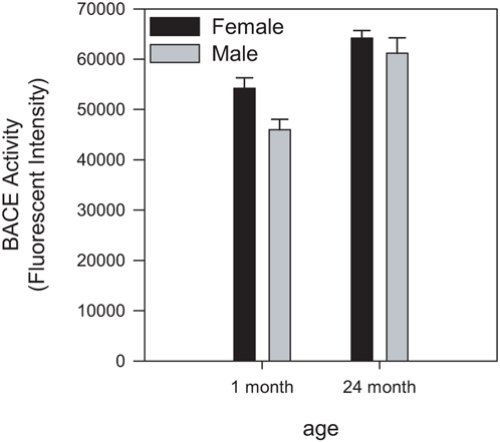
Analysis of BACE1 activity in young and aged C57BL/6 mouse brains. Both male and female mice show an increase in BACE1 activity in aged brain. 10 µg brain membrane was assayed for BACE1 activity using a 12-mer FRET based substrate. Background was defined as activity remaining in the presence of 5–10 µM StatineVal. Values represent background subtracted fluorescent intensity (average±s.e.m., n = 14).

We then examined BACE1 activity in young (1 month) and aged (24 month) brain. Membrane isolated from mouse brain was incubated with an APP peptide substrate containing the Swedish mutation in the presence or absence of StatineV, a potent BACE1 inhibitor. Background was defined as the activity remaining in the presence of StatineV. At 1 month of age, female mice had about 15% more BACE1 activity than male mice (Figure 2, p<0.009). However, we found that both male and female mice had a 20–30% increase in BACE activity at 24 months compared to 1 month (Figure 2, p<0.0005). Significantly, there was no gender associated difference in BACE1 activity in aged brain (Figure 2, 24 months, p = 0.4).

**Figure 3:**
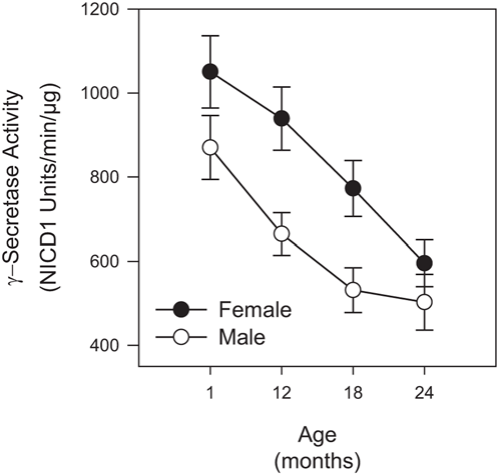
Age- and gender-specific Notch1 γ-secretase activity in C57BL/6 mouse brain. Both male and female mice exhibit an age-dependent reduction in Notch1 γ-secretase activity. *In vitro* γ-secretase activity for NICD1 production from total mouse brain was measured using a recombinant Notch1 substrate. Background was defined as activity remaining in the presence of 1 µM Compound E. Values represent background subtracted ECL units/min/µg membrane (average±s.e.m., n = 16).

In addition to APP, γ-secretase is also known to process Notch1. Therefore, we wished to determine if there were any age or gender dependent changes in γ-secretase activity for Notch1 processing as we saw with APP processing. Total whole brain membrane fractions from 1, 12, 18, and 24 month old C57BL/6 mice were assayed for *in vitro* Notch1 γ-secretase activity using a recombinant Notch1 substrate. Both male and female mice had similar levels of Notch1 activity at 1 month of age, producing about 1000 NICD1 units/min/µg (Figure 3, p = 0.1). However, Notch1 γ-secretase activity declined sharply with age; both male and female aged mice (24 month) exhibited only about 50% of the Notch1 activity as compared to young mice (1 month) (Figure 3, Female p<0.0002, Male p<0.001). While female mice had higher Notch1 γ-secretase activity at 12 and 18 months compared to males (p<0.006 and p<0.01, respectively), there was no gender dependent differences in Notch1 activity at 24 months (p = 0.3).

### Both male and female aged brains have reduced levels of total Nct and PS1

**Figure 4:**
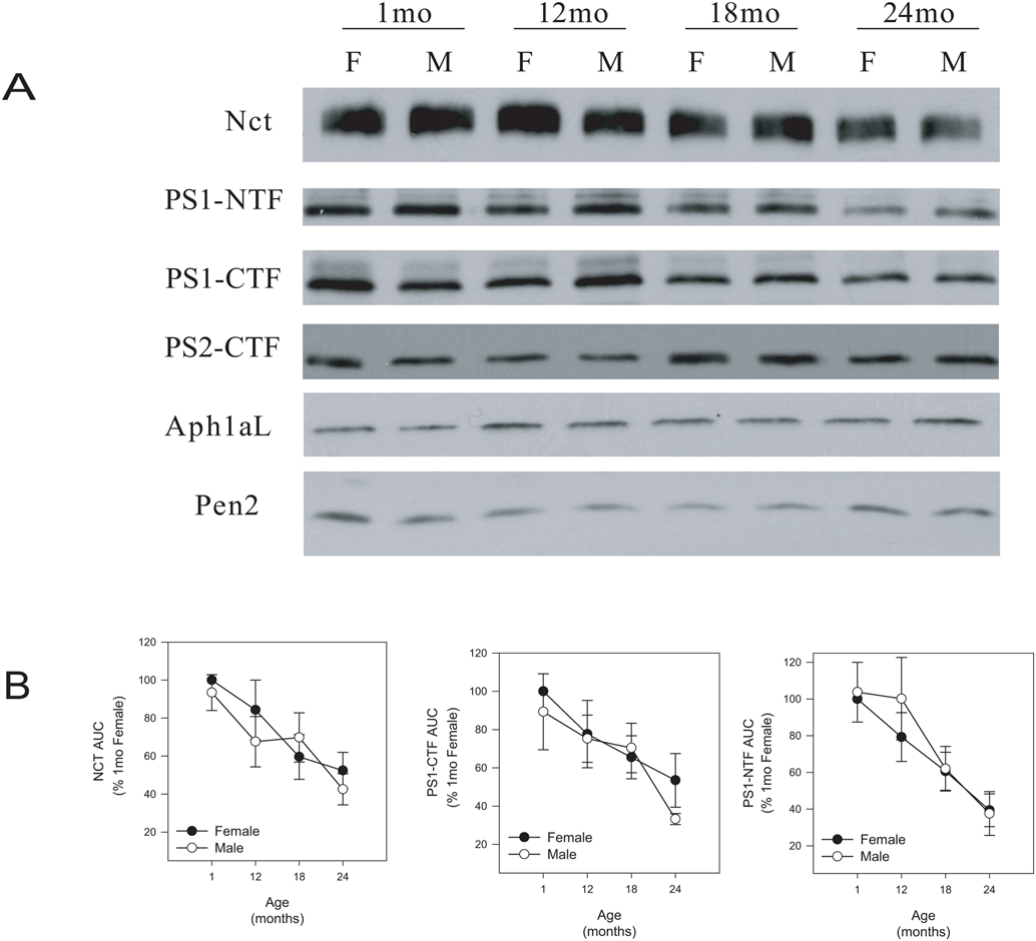
Western blot analysis of total protein levels of γ-secretase subunits in C57BL/6 mouse brain. (A) Both female and male mice have a reduction in the protein levels of Nct and PS1, but not PS2, Aph1aL or Pen2, in aged brain. Equal amounts of total brain membrane was separated on SDS-PAGE and western blotted for the indicated subunits of γ-secretase. Representative blots shown. F = Female, M = Male. (B) There is a 50–60% reduction in total protein levels of Nct (left) and PS (middle and right) in 24 month male (open circles) and female mice (filled circles). Quantification of the relative band intensities of multiple western blots was calculated by measuring the area under the curve (AUC) using the gel analysis function of ImageJ software. Blots were internally normalized to the relative band intensity of 1 month female. Values represent percentage of 1 month (%1 mo) female AUC. (average±s.e.m. n = 4).

Because there was a significant difference in γ-secretase activity between male and female brain at 24 months, we determined if there were any changes in the protein levels of the core subunits of γ-secretase which could account for the distinct sex specific activity profiles. Equal amounts of total membrane were resolved by SDS-PAGE and western blotted for the core γ-secretase subunits: nicastrin (Nct), presenilin (PS), Aph1aL, and Pen2. There was a reduction in Nct and PS1 levels at 24 months in male and female mice (Figure 4A, panels 1–3) but no change in expression of PS2-CTF, Aph1aL, and Pen2 with aging (Figure 4A, panels 4–6). The area under the curve (AUC) was measured from multiple western blots and normalized as a percentage of 1 month female as a means of quantification. There was a significant 50–60% reduction in the levels of Nct, PS1-NTF, and PS1-CTF for both female and male mice at 24 months (Figure 4B, p<0.03). There were no significant changes in the protein levels of PS2-CTF, Aph1aL, and Pen2 (data not shown).

### Male, but not female, mice show reduced photolabeling of presenilin in aged brain

**Figure 5:**
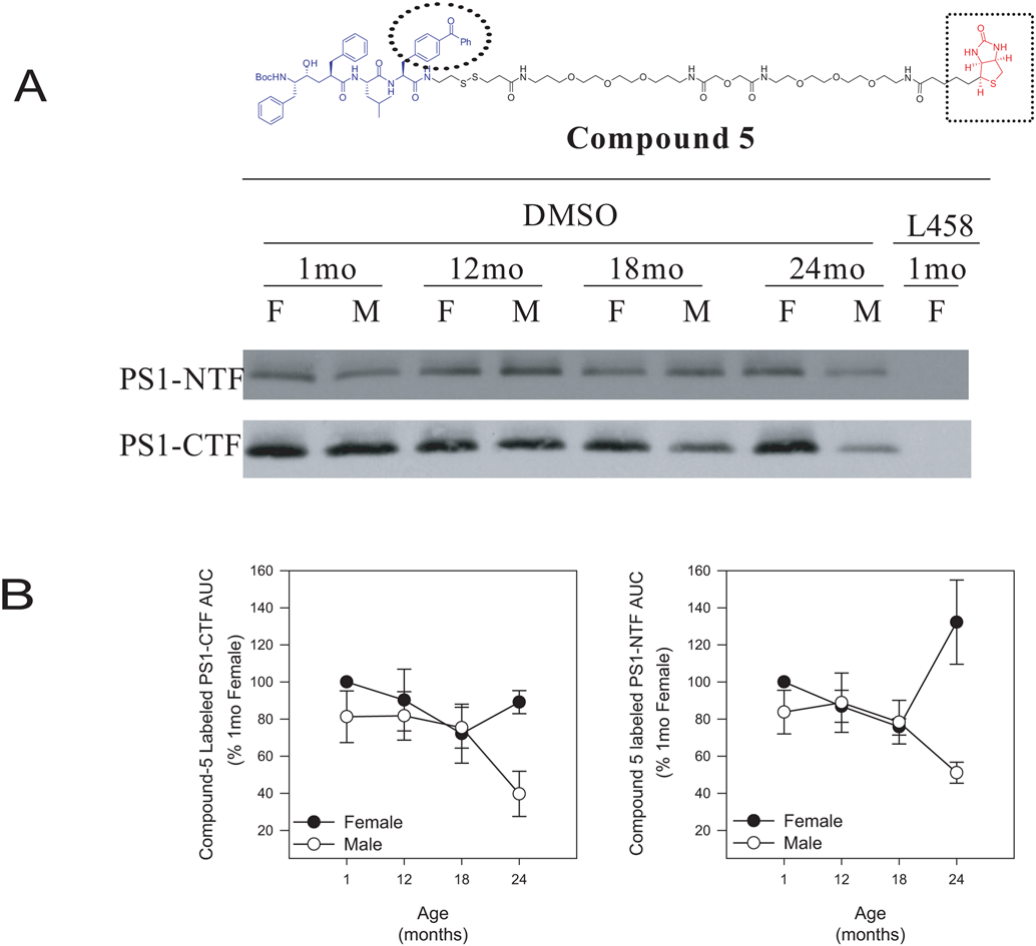
γ-Secretase active site photoloabeling of C57BL/6 mouse brain. (A) Aged male mice have reduced photolabeling of PS1 compared to female mice at 24 months. The structure of compound 5 is indicated. The biotin moiety is marked with a dashed box, the photoreactive benzophenone is marked with a dashed circle, and the linear distance between the biotin moiety and the L458 backbone is 57Å as determined in ChemDraw. Equal amounts of total membrane were covalently photolabeled with compound 5 in the presence (L458, 1 month female shown as representative) or absence (DMSO) of non-biotinylated L458. Following denaturation with RIPA buffer, the labeled proteins were captured with streptavidin agarose, separated by SDS-PAGE and western blotted for PS1-NTF (upper panel) and PS1-CTF (lower panel). Representative blot shown. F = Female. M = Male. (B) Male mice have a 60% reduction in PS photolabeling at 24 months compared to females. Quantification of the relative band intensities of multiple photolabeling experiments was calculated by measuring the area under the curve (AUC) using the gel analysis function of ImageJ software. Blots were internally normalized to the relative band intensity of 1 month female. Values represent percentage of 1 month (%1 mo) female AUC. (average±s.e.m., n = 4).

We have previously shown that γ-secretase activity does not always correlate with total expressed subunit levels [25]. In addition, only a fraction of expressed subunits, as measured by western blotting, are actually incorporated into enzymatically active complexes [26], [27]. Therefore, western blotting alone is not adequate for exploring the relationship between activity and expression of active complexes. We previously developed and characterized a panel of small molecule affinity probes based on the L458 backbone which can be used to study the composition of active γ-secretase complexes [25]. We designed an improved version, designated compound 5, with a longer and more hydrophilic biotin linker for capture of endogenous active γ-secretase and a photoreactive benzophenone group that can be used to covalently photolabel the active site (Figure 5A). Compound 5 can inhibit γ-secretase isolated from HeLa membrane with an IC50 of ∼4 nM (data not shown). We used compound 5 to photolabel PS1-NTF and PS1-CTF, the catalytic core, of γ-secretase. Total brain membrane was incubated with compound 5 in the presence or absence of excess L458 and irradiated to covalently crosslink the probe with the active site of γ-secretase. The labeled components were separated by SDS-PAGE and western blotted for PS1-NTF and PS1-CTF. The catalytic core was clearly photolabeled by compound 5 and this labeling was completely blocked by inclusion of excess L458, indicating specific labeling of the target protein. Male mice exhibited a reduction in photolabeling of both PS1-NTF and PS1-CTF at 24 months (Figure 5A, M = male). However, female mice did not display the same decrease in photolabeling at 24 months as male mice (Figure 5A, F = female). Quantification of the relative AUC of multiple photolabeling experiments revealed male mice had approximately a 45% reduction in PS1-CTF and PS1-NTF photolabeling at 24 months compared to 1 month (Figure 5B, open circles) whereas female mice had no significant changes in the amount of labeled PS1 (Figure 5B, filled circles). At 24 months male mice had 60% less photolabeled PS compared to female mice, indicating that male mice had less active γ-secretase complexes than female mice in aged brain or an altered conformation of the active site (Figure 5B, p<0.016).

## Discussion

**Figure 6:**
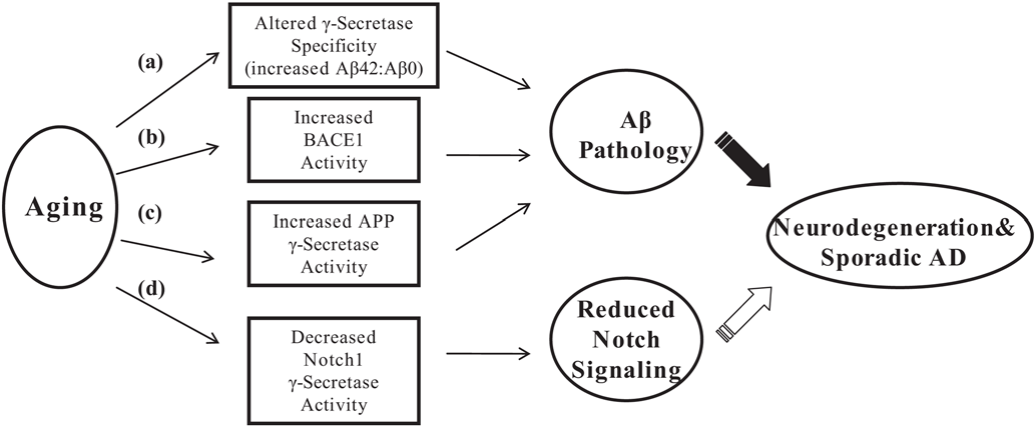
Model of potential age and gender dependent pathogenic pathways resulting in neurodegeneration. Pathologic Aβ production can be mediated by (a) altered γ-secretase specificity resulting in increased Aβ42∶Aβ40 ratios leading to increased pathogenic plaque deposition (b) increased BACE1 activity which increases the levels of available APP substrate and therefore production of Aβ peptides (c) increased APP γ-secretase activity which results in increased Aβ production. In addition to pathogenic Aβ pathways, loss of Notch1 signaling due to decreased Notch1 γ-secretase activity may also lead to a neurodegenerative state (d). The data presented here implicates aging in pathways (a), (b), and (d) in the development of AD whereas (c) is an additional causative pathway implicated in the pathogenesis of neurodegeneration in females.

Aging and gender are risk factors that are clearly associated with sporadic AD. However, whether or not these risk factors directly affect γ-secretase production of Aβ is not known. Multiple mouse models have been used to recapitulate Aβ pathology and have proved to be useful tools in understanding the pathogenesis of AD [28]. We therefore utilized wild type mouse brain to examine the effect of age and gender on the activity and specificity of γ-secretase for Aβ production. First, we found an increased Aβ42∶Aβ40 ratio in aging mice indicating that γ-secretase specificity for Aβ40 and Aβ42 processing varies with age. Recent studies suggest that the ratio of Aβ42∶Aβ40, rather than the total levels of Aβ, is better correlated with AD pathology and subtle changes to the Aβ42∶Aβ40 ratio promotes Aβ aggregation and toxicity [29]–[31]. Therefore, our data which demonstrates increased Aβ42∶Aβ40 ratios in aged mice may account for, in part, the increased incidence of sporadic AD seen during aging (Figure 6, (a)). In addition, we found that aging also leads to an elevation of BACE1 activity, which is consistent to previous reports showing increased BACE1 activity in both aged and AD brains and further supports a role for aberrant APP processing in sporadic AD (Figure 6, (b)) [21], [22]. Second, we have shown that aged female mouse brain has 65% more *in vitro* γ-secretase activity at 24 months compared to male mice. Although female and male mice both have increased Aβ42∶Aβ40 ratios at 24 months, the fact that female mice have elevated γ-secretase activity suggests that their overall plaque burden would be increased compared to male mice due to increased APP processing (Figure 6, (c)). Furthermore, we demonstrated that there were no gender differences in BACE1 activity in aged brain, indicating that the increased *in vitro* γ-secretase activity in female mice at 24 months would likely be translated into increased Aβ production *in vivo*. These data establish a potential role for elevated APP γ-secretase activity in aged females as an additional causative factor in the increased prevalence of AD in women and increased plaque burden seen in female mouse models of AD.

Photolabeling of PS1 suggests that the reduction in γ-secretase activity seen in aged males is due to either the reduced formation of active complexes or alterations to the active site architecture which reduce APP processing. However, the precise mechanism by which high levels of γ-secretase activity are maintained in aged female mouse brain remains unknown.

The decline in estrogen during menopause has been suggested to be a causative factor in the increased prevalence of AD in women. Early reports of hormone replacement therapy suggested a protective role of estrogen in the pathogenesis of AD [32]–[34]. However, more recent reports have found that estrogen replacement fails to protect against the development or progression of AD and in some cases worsens disease progression [35]–[38]. Estrogen has been shown to reduce APP processing in animal and cell model systems via a variety of mechanisms including increased trafficking of APP out of the trans-Golgi network and increased α-secretase mediated APP cleavage which would lead to a reduction in available APP substrate for γ-secretase processing [39]–[41]. Estrogen deficiencies have been associated with increased Aβ deposition via increased BACE1 activity and decreased neprilysin/insulin-degrading enzyme activity, both of which were detected as gender specific biochemical markers associated with aggressive plaque deposition in mouse models of AD [12], [13], [42]. It is known that changes in estrogen levels can influence the lipid and lipoprotein profiles in women [43]–[46]. Furthermore, it is known that changes to the lipid microenvironment can have a large impact on γ-secretase activity [47]–[49]. A cellular model of aging in normal human fibroblasts demonstrated a downregulation of γ-secretase activity due to disruptions in the lipid raft microenvironment [50]. Therefore, the potential effect of aging and estrogen deficiencies on the lipid environment of brain and γ-secretase activity warrants investigation and may provide insight into the gender specific γ-secretase activity differences in aged brain we observed.

Interestingly, the gender dependant effects on γ-secretase activity appear to be specific for APP processing as both male and female mice exhibited a decline in Notch1 γ-secretase activity during aging and exhibit no gender associated differences at 24 months. Aged brain γ-secretase complexes, therefore, have gender dependent substrate processing abilities with female aged brain strongly favoring APP over Notch1 as a substrate and male brain showing no significant age related changes in its relative substrate preference. These data suggest that discrete γ-secretase complexes exist in brain which exhibit unique activity profiles, substrate processing abilities and regulation.

Notch signaling pathways are critical for cell fate decisions during development and direct proliferative, differentiation, and apoptotic responses [51]. In addition to its pivotal role in neurogensis during embryonic development, Notch signaling is involved in many aspects of central nervous system development in the postnatal and adult brain including the maintenance of neuronal stem cells, neuronal proliferation, and dendritic arborization; this Notch signaling is dependent on PS expression and function [52]–[55]. Importantly, it has been shown that a subset of PS FAD mutations result in the loss of γ-secretase mediated Notch1 cleavage [56]–[58]. Furthermore, conditional loss of PS in adult mouse brain leads to loss of synaptic plasticity, memory impairments, and age-dependant neurodegeneration through the loss of Notch activation of CRE-dependent gene expression [59]. Therefore, the age dependent reduction in Notch1 γ-secretase activity we detected in male and female brain may also contribute to the severity of sporadic AD due to enhanced neurodegeneration (Figure 6, (d)).

In summary, our data implicate a role for altered γ-secretase activity during aging in the development of sporadic AD and neurodegeneration; aged mice exhibit altered specificity for Aβ40 and Aβ42 production and decreased Notch1 processing, both of which are reminiscent of certain PS FAD mutations that result in the development of early onset AD. Furthermore, our demonstration that female mice have much greater γ-secretase activity in aged brain compared to males offers an additional model to explain the increased prevalence of AD in women and aggressive plaque pathology seen in female mouse models. Increased γ-secretase activity, along with altered Aβ42 and Aβ40 specificity, in aged brain, would potentially exacerbate the plaque load in females. This model would work in concert with previously demonstrated increases in BACE1 activity and reductions in neprilysin activity in female mouse models of AD. Additionally, loss of Notch1 γ-secretase activity in aged mouse brain may also augment the pathological consequences of increased Aβ42∶Aβ40 ratios and increased Aβ production by increasing neurodegeneration due to the loss of activated Notch1 mediated CRE-dependent gene expression. Therefore, our data help to clarify the roles of aging and female gender as risk factors for sporadic AD and advance our understanding of aberrant γ-secretase activity as a potential mechanism underlying the pathogenesis of sporadic AD.

## Materials and Methods

### Chemical compounds and antibodies

Synthesis of L685,458 and Compound E were previously described [60], . Compound 5 was synthesized by using the same procedure as previously described [62]. Nct and SM320 (activated Notch1) antibodies were generated in our laboratory. Antibodies against PS1-NTF and Pen2 were a kind gift of Dr. Min-tain Lai (Merck Research Labratories) and Dr. Jan Näslund (Karolinka Institutet), respectively. The remaining antibodies were commercially purchased: PS1-CTF (Chemicon, MAB5232), PS2-CTF (Calbiochem, PC235), and Aph1aL (Zymed, 38–3600). H-Glu-Val-Asn-[(2R,4S,5S)-5-amino-4-hydroxy-2,7-dimethyl-octanoyl]-Ala-Glu-Phe-OH (StatineV) was purchased from Bachem.

### Membrane preparation

C57BL/6 mouse whole brain (male and female at 1, 12, 18, and 24 months of age) was obtained flash frozen from the NIA/NIH aged rodent tissue bank (Bethesda, MD) or special ordered from Taconic (Hudson, NY). Animals were tumor free with no gross pathologies or illnesses. Total membrane was isolated from two brains for each age and gender (Table S1). Briefly, 2 whole brains were thawed on ice, resuspended together in ice cold Buffer A (50 mM MES, pH 6.0, 150 mM KCl, 5 mM CaCl_2_, 5 mM MgCl_2_, and protease inhibitors), and Dounce homogenized. Nuclear debris was cleared by low speed centrifugation and the resulting supernant was ultracentrifuged 100,000×g for 1 h at 4°C. The resulting pellet, representing the total membrane fraction, was resuspended in Buffer A and Dounce homogenized. Protein concentration was measured with the DC Protein Assay Kit per manufacturer's instructions (Bio-Rad) and was approximately 5 mg/mL.

### *In Vitro* γ-Secretase Activity Assay

γ-Secretase activity was measured using the previously described electrochemiluminescence method [63], [64]. Brain membrane (4 µg) was incubated with Buffer B (50 mM PIPES pH 7.0, 150 mM KCl, 5 mM CaCl_2_, 5 mM MgCl_2_ and protease inhibitors) with 0.25% CHAPSO (v/v), 1 µM recombinant biotinylated APP substrate or 0.2 µM recombinant biotinylated Notch1 substrate, and 0.1% bovine serum albumin (v/v) in the presence of 1 µM Compound E or DMSO for 2.5 h at 37°C. The reaction mixture was incubated with ruthenylated G2-10 or G2-11 to detect Aβ40 and Aβ42 peptides, respectively, or SM320 in conjunction with ruthenylated anti-rabbit to detect Notch1 intracellular domain (NICD1) in Buffer C (1× phosphate-buffered saline and 0.5% Tween 20 (v/v)) for 2 h at room temperature. Immunocomplexes were captured with magnetic strepavidin beads (Dynal) and Aβ40, Aβ42, and NICD1 production was measured by electrochemiluminescence. Activity is expressed as relative light units/min/⋯g membrane. Brain γ-secretase activity was measured from two independent membrane preparations (n = 4 animals total). Data points from four independent assays (2 assays for each individual membrane preparation) were averaged. P values were determined using a student t test in SigmaPlot8.0.

### *In Vitro* BACE1 Activity Assay

BACE1 activity was measured using a 12-mer FRET based substrate (TAMRA-Glu-Glu-Ile-Ser-Glu-Val-Asn-Leu-Asp-Ala-Glu-Phe-QSY 7 amide). 10 µg membrane was incubated in BACE1 Assay Buffer (500 mM Sodium Acetate pH 4.5, 150 mM NaCl, and 0.1 mg/mL BSA) with 0.2% CHAPS (v/v) and 5 µM substrate in the presence or absence of 5–10 µM StatineV for 2 h at 37°C in the dark. BACE1 cleavage of the substrate was detected by excitation of the fluorophore at 530 nm and emission was monitored at 580 nm using a 50/50 beam splitting mirror (Envision, PerkinElmer). Activity is expressed as fluorescent intensity at 580 nm. Activity was measured in two independent membrane preparations (n = 4 animals total). Data points from two independent assays were averaged. P values were determined using a student t test in SigmaPlot8.0.

### Western blotting and photolabeling

For western blotting, 20 µg total membrane was prepared with 4× reducing Lammeli sample buffer, separated by SDS-PAGE and transferred to PVDF membrane. Following blocking, the following antibodies were used for western blotting: Nct (1∶1000), PS1-NTF (1∶1000), PS1-CTF (1∶1000), PS2-CTF (1∶1000), Aph1aL (1∶250), and Pen2 (1∶500). Anti-mouse or anti-rabbit horseradish peroxidase conjugated secondary antibodies were used in concert with standard enhanced chemiluminescence detection methods. For photolabeling, 800 µg total membrane was pre-incubated with 2 µM non-biotinylated L458 or DMSO for 0.5 h at 37°C in Buffer B supplemented with 0.25% CHAPSO (v/v). Following pre-incubation, 20 nM compound 5 was added for 1 h at 37°C. Covalent crosslinking was performed by photoactivation of the benzophenone moiety at 350 nm for 0.75 h on ice. The reaction was denatured with 1× RIPA (50 mM Tris pH 8.0, 150 mM NaCl, 0.1% SDS (w/v), 1% Nonidet P40 (v/v), and 0.5% deoxycholic acid (w/v)) for 1 h at room temperature. The labeled complexes were captured with streptavidin agarose for 16 h at 4°C. The captured complexes were washed with 1× RIPA, eluted with 2× reducing Lammeli sample buffer, and resolved by SDS-PAGE. Western blotting was performed as described above. In all cases, blots are representative of a minimum of three independent experiments.

## Supporting Information

Table S1 (0.78 MB EPS)
